# A Psychoneuroimmunological Reading of Jane Austen’s *Persuasion* in the Context of Bodily Aging

**DOI:** 10.1007/s10912-024-09845-1

**Published:** 2024-04-05

**Authors:** Rocío Riestra-Camacho, Miguel Ángel Jordán Enamorado

**Affiliations:** 1 Department of English, French and German, University of Oviedo, Oviedo, Spain; 2 Department of English Studies, University of Valencia, Valencia, Spain

**Keywords:** Jane Austen, *Persuasion*, English literature, Body, Aging, Psychoneuroimmunology

## Abstract

Jane Austen normally avoids discussing appearance throughout her works. *Persuasion* constitutes the exception to the rule, as the story focuses on the premature aging experienced by her protagonist, Anne Elliot, seemingly due to disappointed love. Much has been written about Anne’s “loss of bloom,” but never from the perspective of psychoneuroimmunology, the field that researches the interrelation between psychological processes and the nervous and immune systems. In this paper, we adopt a perspective of psychoneuroimmunology to argue that Austen established a connection between psychological distress, specifically lovesickness, and the development of early senescence signs, and vice versa, since the recovery of love is associated with happiness and physical glow. From a gender perspective, we discuss how Austen brightly reflected these interrelationships through the story of Anne, when the latest psychoneuroimmunological research has actually shown that women age earlier than men as a consequence of psychological turmoil.

## Introduction: Medicine and literature

The study of novels as a source of information for research in the field of health is a “fairly unexplored area” (Kaptein 2021a, 3). There are various approaches to this issue. On the one hand, we can find studies on the medical history of some authors and their possible influence on their novels, such as the research by Wilson on Jane Austen’s eye pathology. We also find studies in which the way in which a disease is represented is addressed in greater or lesser depth. Some of them focus on a specific work, as is the case of Zayas et al. (2007), who analyzed the representation of migraine in the novel *The Master and Margarita* by Bulgakov, or Wilkinson’s (2019) study on melancholy in *Hamlet*. Others open their point of view to universal literature, as occurs in Wolf’s (1995) work on epilepsy or Kaptein’s (2021b) on cancer. There are also scholars who focus on particular authors, such as Del Guercio (2019), who analyses psychopathology in the works of Edgar Allan Poe. Finally, studies can also be found that compare the literary representation of some diseases with the empirical image of clinical histories of said disease, as would be the case of the research carried out by Florijn et al. (2018) on prostate cancer.

In this paper, we will approach the relationship between literature, medicine, and psychology from an innovative perspective. In it, we will focus on exposing some features of Jane Austen’s work *Persuasion* in the context of the psychoneuroimmunology field. We will expose how Jane Austen elucidated the relationship between mental health and physical appearance—and more specifically between psychological distress—represented through the “sickness of love” of her protagonist and having a worn physical appearance, which Austen captured in the representation of “loss of bloom” by Anne Elliot.

## Romanticism and neuroscience

The advancement of physiology in the seventeenth and eighteenth centuries caused the relationship between the mind and the body to be seen from two opposite perspectives at the same time. Specifically, the classical conception of a dualistic nature, which followed the traditional belief of the soul-body division, was joined by the proposal that aligned mental acts with brain functions, open to a materialist interpretation, closer, in fact, to the current vision of psychoneuroimmunology (Richardson 2001, 1).

When approaching the psychology of Romanticism from the perspective of current knowledge in the field of neuroscience, it is remarkable to discover how ahead of their time were the studies and experiments on the brain carried out by scientists and the way these issues were dealt with by some literary authors of the time. The intersection between science and literature during Romanticism justifies the fact that the history of neuroscience has rediscovered this period in recent decades, in which a cognitive revolution has occurred with numerous investigations on the relationship between the mind, the emotional system, and the body. In particular, current neuroscience focuses on something that was only beginning to be glimpsed at the time of Romanticism: that the relationships between brain and body and between body and brain are bidirectional and that, in reality, we cannot separate one entity from another, something that Romantic literature managed to capture on paper in a revolutionary way (Lau 2018).

Graham Richards (1992), in his landmark study of psychological ideas, highlighted the important contribution of early nineteenth-century novels in delineating the “new individuality” associated with contemporary advances in physiological psychology. Similarly, he credited Romantic poetry with revitalizing psychological language and creating new ways of articulating, developing, and evaluating subjective experience.

We find in British Romanticism a common desire of artists and scientists to investigate the relationship between mind and body. In fact, the novel of this time had begun to adopt and even extend the brain-centered studies of mind and personality that had been popularized by the phrenology of the day in its debates on current materialist and vitalist theories. The novels and poetry of female writers, despite the inequality in their education, were not alien to the scientific culture of their time, and therefore, we also find a reflection of these issues in their works, and without a doubt, the novels by Jane Austen are a paradigmatic example (Richardson 2002, 94).

As will be explained later, Austen anticipates the Victorian novel by speaking in her works of the biological and innate traits of the mind and character of her protagonists, following the postulates of the “neuroscience” of her time and, on occasions, even anticipating them with her psychological intuition (Richardson 2002, 142). The study of thoughts and emotions that Austen carries out in her novels reaches a higher level of psychological appreciation in *Persuasion* by focusing on embodied cognition and unconscious mental processes as a new way of representing conscious experience (Richardson 2002, 94). We can say that Austen understands the mind and body as intrinsically interrelated entities and in a flow of constant interaction.

### Knowledge about the mind in the regency era

The mental health problems of King George III, leading up to the Regency period, sparked widespread interest in mental illness in the early nineteenth century (Bewley 2008). However, it took several decades for significant advances to take place in this field. Medical knowledge during the Regency was still very limited. Antiseptics were unknown, as was the existence of germs, which was demonstrated in 1854 when the cholera epidemic was linked to contaminated water sources (Brody et al. 1854). The idea that the brain played a fundamental role in mental functions was introduced in the mid-eighteenth century by the British philosopher David Hartley and the French scientist Julien Offray de la Mettrie and developed in the early nineteenth century by the German Franz Joseph Gall (Schacter et al. 2011).

Due to its unobservability, the mind was thought to be “incapable of disease or of its final consequence, death” (Gray 1885, 46). However, the mind could suffer a variety of disorders and, therefore, should be treated in the only possible way, that is, through the body, since the mental disorder reflected the sympathy of the mind with some state of bodily disorder (Arieno 1989). Therefore, said mental disorder was treated according to the underlying physical illness to which it was linked, following the procedure proposed by the current of Humoralism, according to which changes in diet and habits had to be implemented to balance the humors and restore health (Waller 2002).

In essence, the second half of the eighteenth century brought with it a “neurocentric” conception of mental disorders, according to which emotional disorders were seen as alterations of the nervous system, which specifically followed the vitalist conception of the time. From a sociological perspective, on the other hand, the shortcomings in the scientific training of people dedicated to medicine had the consequence that the diagnosis could be marked by the socioeconomic characteristics of the patient. For this reason, a disease such as scurvy was attributed to a lack of personal hygiene and an insufficient diet, if the patient belonged to the working class, or to the excess of difficult-to-digest foods, if it was a wealthy person (Waller 2002).

## Jane Austen and health

Austen’s novels lack physical descriptions. Throughout her work, the author rarely stops to inform readers of the protagonists’ appearance beyond some vague reference to their physical attractiveness. However, the body and, specifically, the female body is present in all her works, but not as a possible object of desire, but in the context of health and disease (Wiltshire 1992, 126).

Health, in Austen’s time, was for women a luxury that should not be risked since any impairment in physical appearance could affect their commercial value in the marriage market, as can be seen in John Dashwood’s comment to Elinor in *Sense and Sensibility* about how Marianne’s illness will mean that she cannot expect a good offer of marriage. In this context, Austen confers great importance on her heroine’s health—be it the strength of the protagonist who gives her name to the novel *Emma*, the weakness of Fanny (*Mansfield Park*), or the loss of bloom of Anne Elliot (*Persuasion*)—since it is linked to their personality, and furthermore, it is a very important element in their social context. Emma’s health enables her to exercise her power at Highbury, Fanny’s frailty is coupled with her vital insecurity, and Anne’s premature aging reflects her suffering.

During the eighteenth century, novels of sensibility were very successful, in which the importance of feelings, sentimentality, and sensibility was highlighted from a perspective very focused on the “alterable” physiology of their protagonists. These works are filled with emotion and, frequently, with exalted feelings that entail physical reactions from their protagonists, such as fainting, anxiety crises, or a livid appearance. The creators of these novels, including Smollett, Goethe, Sterne, and Richardson, out of others, exaggerated the relationship between physiology and feelings, which lies at the core of this genre (Gorman 1990). Austen read novels of sensibility that, in fact, she parodied in her youth writings, in which the protagonists fell unconscious in turns or felt themselves dying due to their amorous passion, which had arisen after a brief encounter with a gentleman (Austen 2006).

Austen’s reaction to this genre follows the line of Charlotte Lenox, who, in her work *The Female Quixote*, attacked the illusions caused by excess sensitivity and the connection of these excesses with hypochondria, hysteria, and fainting. A clear example of the pathological consequences of this self-referential emotion is hypochondria, which was known as “The English Malady” and which we find present in Austen’s work through characters such as Mrs. Bennet, Mr. Woodhouse, Mrs. Bertram, or Mary Musgrove (Kenyon 1965). Austen’s disapproval and criticism of hypochondria is a consequence of her personal experience in nursing her mother. Numerous references to Mrs. Austen’s delicate and variable situation are found in Austen’s letters (Takei 2005, 149). The author herself did not enjoy good health, as she suffered from uncomfortable eye and neuralgic pathologies, which undoubtedly could have influenced the importance given to health and the neural issues in her protagonists (Takei 2005, 149).

## Jane Austen and the mind

Austen’s literary style is characterized, among other features, by her ability to realistically and profoundly represent the psychology of her characters (Jordán 2017). This fact is due in part to her ability to observe and her constant study of personality but also to the influence of some of the authors she read, such as William Shakespeare, James Boswell, or Elizabeth Hamilton (Kennedy 2012).

In speaking of madness, Boswell described it as a deviation from the usual ways of the world, which could be partial and intermittent and whose cause could be found in an excess of imagination, illness, or distressing events (Boswell 1890, 487). Austen read Boswell’s work, and his influence can be seen in various passages in her novels, as, for example, in Captain Benwick’s thought process in *Persuasion*, which changes from extreme melancholy and excessive sensitivity to poetry as a consequence of the death of his beloved to a new crush and marriage in just a few months (Overmann 2013, 121). For her part, Fanny Price experiences “feelings of sickness” that make her go “from hot fits of fever to cold” upon hearing the news of the adulterous behavior of Mr. Crawford and Mrs. Rushworth (Austen 1833, 394). That is, the emotional disturbance affects the mind and manifests itself in the body. In this way, Austen reflects the dominant medical opinion that immaterial mental disorders must be treated through the materiality of the body (Arieno 1989). Fanny prevents her mind from falling into a deeper state of melancholy, which could lead her to psychosomatize her suffering through physical activity, proving the effectiveness of “employment, active, indispensable employment, for relieving sorrow” (Austen 1833, 396).

In *Sense and Sensibility*, we find an in-depth analysis of mental balance through the study of Elinor and Marianne Dashwood. Elizabeth Hamilton defines mental equilibrium as the fact of “unit[ing], or rather blend[ing], two distinct principles of our nature—the affections of the heart, and the faculties of the understanding” (Kennedy 2012, 229). From this point of view, it could be said that *Sense and Sensibility* is the novel in which Austen deals most carefully with the mind and madness. Speaking of madness, we refer here to the period in which the heart and understanding of both sisters are torn by disappointed love. Marianne’s madness, more severe than Elinor’s, necessitates the transition to a physical illness in order to heal, following the process that was considered natural in Austen’s time (Overmann 2013, 122). Elinor, however, is able to dominate the psychosomatic manifestation of her suffering through self-control and thus avoid the bodily effects of love pain (Tavela 2017, 134).

The reason for heartbreak and its influence on health that we will focus on here is therefore not exclusive to *Persuasion*, but it has been said that this novel is unique in that it “remains the only Austen novel in which facial color (bloom) alters as relationships change and love is recovered,” one phenomenon to consider from a psychoneuroimmunological perspective (Gorman 1990, 249).

## Psychoneuroimmunology: A brief introduction

It is a common belief that sleeping well improves the look of people’s faces, whereas stress makes them appear cranky and prone to catching illnesses, or that pregnant women and couples in love have a certain “glow,” while subjects who have gone through psychological turmoil may look older than their actual age. In the 1970s, these phenomena started to be studied from a scientific perspective. Once the field of “psychoneuroimmunology” opened up, researchers were able to begin articulating the common factors underlying such different physiological variations humans experience. Psychoneuroimmunology, specifically, is the discipline specializing in the study of the nervous system, the endocrine system, the immune system, and the psychological factors that underlie their interactions.

The nervous system comprises the brain, the spinal cord, and the nerves, and it is considered the system that coordinates an organism’s actions and sensory information by transmitting signals to and from different parts of the body. The endocrine system, on the other hand, is made up of the body’s hormones and regulates biological and metabolic processes. In vertebrates, the immune system is made up of the lymphatic system, which, in humans, includes organs and tissues such as the thymus or the bone marrow. This latter organ is the one that produces immune cells. The system functions as a network of interrelated biological processes that protect an organism from disease.

Before the implementation of psychoneuroimmunology and throughout the 1950s, the immune system was considered a defense mechanism that protected the organism autonomously. This point of view started to prove insufficient when it was discovered that the immune system was affected by stress. In particular, individuals under strain were more susceptible to infections (Goodkin and Visser 2000, 57). Psychological aspects have hitherto come to be considered as factors underlying the functioning of the immune system—for good and for bad.

Essentially, when the immune system is attacked, it activates itself in an attempt to restore balance, which is normally referred to as “homeostasis” in reference to an organism preserving a similar (“homo”) physical and chemical state (“stasis”) as prior to the attack. In order to maintain this equilibrium, the immune system is in constant interrelation with the other two systems, the autonomous system and the endocrine system, and each of these can influence the state of the other. On many occasions, a psychologically relevant event acts as a trigger that kick-starts this kind of interaction between the systems.

Cytokines, a specialized group of chemicals released by these three systems, play a vital role in this tripartite interaction. Their function is to coordinate different physiological and psychological processes involved in maintaining organic homeostasis. During illness, cytokines regulate many physiological reactions, such as fever, but they also affect organisms’ behaviors. For example, throughout an infectious process, the release of cytokines induces symptoms of fatigue, making a subject prioritize rest over activity in order to favor recovery (Dantzer et al. 2001).

The impact cytokines have on health and disease is also patent in mental illnesses, including depression, anxiety disorders, schizophrenia, and addictions. By way of illustration, subjects with depression present lower levels of certain cytokines, namely lymphocytes and killer cells, in comparison to healthy cohorts. Reduced killer cell levels are even more pronounced when depressive symptoms are accompanied by stress. This could account for the fact that depressed people are more prone to catching infectious diseases (Reiche, Nunes, and Marimoto 2004, 618).

This insight about the activity of cytokines serves to illustrate the complexity of health processes, as well as to show how interrelated physiological and mental health processes can be. In essence, the field of psychoneuroimmunology is interested in looking at the relationship between the body and the mind. This latter clarification is important because dualistic disciplines look at the relationship either between mind and body or between body and mind, but only in that direction, whereas psychoneuroimmunology looks at their two-way interactions, much like the Romantics ventured doing at the time of Jane Austen.

## Premature ageing: The psychoneuroimmunology of Austen

When an organism falls ill, the condition can be the result of the immune system having been exposed to external factors, such as viruses or bacteria, or internal factors, including psychological turmoil. Aging is an excellent case of how both types of phenomena can show on the body, and *Persuasion* elegantly contemplates examples of the two—namely Anne Elliot’s “loss of bloom” as a result of lost love and the aging of sailors as a consequence of inclement weather.

When it comes to aging, it is important to differentiate between chronological age and biological age. Chronological age is the result of the passage of time, and it is a given. On the other hand, biological age manifests in accumulated damage to cells, and it can fall behind or outpace chronological age: one might look younger or older than their actual age. Biological age is, therefore, modifiable, and it can be so due to both external and internal factors.

The skin is one of the best-researched organs in relation to biological aging. Skin aging is defined as “an enhanced process of degradation of skin structural integrity and functionality upon exposure to environmental factors,” causing it to display wrinkles, dyspigmentation, and uneven skin tone (Lee, Watson, and Kleyn 2020, 54). It also leads to sagging and reduced elasticity (Cho et al. 2019, 2). External factors affecting the condition of one’s skin include exposure to different stimuli. For instance, chronic sun exposure has long been known to accelerate skin aging (Lee et al. 2020, 54). However, one of the least researched topics in relation to skin aging is the impact of psychological factors (Lee, Watson, and Kleyn 2020, 57).

Psychoneuroimmunologists have started to consider the possibility that skin aging can be accelerated by negative psychological states. Han et al. (2019, 294–95) claim that biological age indicators “are indeed…sensitive to psychological states, namely stress and psychopathology,” which can directly impact organic tissues like the skin. The impact of stress on the skin is reflected in increased cutaneous inflammation, constituting a possible causal link accounting for sprouts of acne or rosacea (Chen and Lyga 2014, 181). Stress also alters neuropeptide expression on the skin, which would account for the formation of wrinkles (Chen and Lyga 2014, 182). Perceived psychological stress has also been linked to marked weight loss (Molina et al. 2021). Stress-induced weight loss and skin aging can be said to give people what we would call a “faded look.”

Psychological stress, therefore, stands as a firm candidate that partially explains premature aging or the situation whenever a person looks older than their actual age, mostly because of how this process shows on their body, face, and skin (Chen and Lyga 2014, 184). This stress-appearance pathway has only recently begun to be developed, and yet Jane Austen already reflected it through Anne Elliot’s well-known “loss of bloom” in *Persuasion*. But she was not only quite innovative when observing the relationship between the psychological turmoil of the protagonist and her accelerated aging process.

Austen was also a determined novelist who chose a female lead role to articulate this relationship. In *Persuasion*, the author displays a sense of intuition about the gender-specific component of premature senescence, particularly in relation to accelerated aging as a consequence of psychosocial and psychobiological factors. From a psychosocial point of view, there are a number of very specific anxiety-inducing situations that have been shown to influence premature aging that is stress-mediated, namely caregiving for the sick (particularly for ill children) and depression (Cho et al. 2019, 184). These are situations where women get more “representation” than men (Murphy and Byrne 2012).

From a psychobiological perspective, it is known that in women, intrinsic skin aging is strongly influenced by changes in the levels of hormones like estrogen, which flows in naturally higher levels in women than it does in men (Cho et al. 2019, 2). Oxytocin is another hormone that has the power to influence aging. Namely, oxytocin is a neuropeptide that can protect organisms against age-related disorders and potentially be an effective preventive mechanism against skin aging (Cho et al. 2019, 1). Oxytocin levels are not as sex-dependent as estrogen levels; however, a recent study has shown that OXTR (an oxytocin receptor) levels are lower in women than in men, particularly in older ones. The authors of the study conclude that “age-related decline in OXTR expression is sex specific” (7). Oxytocin is popularly known as a “happy hormone” or more specifically as “the love hormone,” since levels of this neuropeptide increase through human contact and bonding, such as when infatuated couples kiss or embrace each other (Ebstein et al. 2012).

The fact that the looks of *Persuasion*’s protagonist change according to her psychological balance, which in turn is made to be dependent on the status of her romantic relationship with Frederick Wentworth, puts Jane Austen somewhat ahead of her time. Even the emphasis placed on the heroine’s role as a caregiver of a sick child, with all the stress that entails for Anne, suggests that Austen sensed the intricate relationship that exists between a woman’s bloom, well-being, and love status.

## Of love and looks in Jane Austen’s *persuasion*

“Anne Elliot desponds at the loss of her first, true love, a rift that has left its mark on her exterior appearance,” claims Jennifer Preston (2021, 189). Much has been written about the marked “loss of bloom” of the protagonist of *Persuasion,* a favorite expression of Austen to refer to Anne’s premature aging. According to Phillips, “bloom,” in Austen’s time, pointed to “a state of great beauty” (Phillips 1970, 80). But it also referred to “healthy facial color, a pinkish glow, a brightness in the eye, the exuberance of youth, and the promise of sexuality” (Gorman 1990, 251). This idea of early deterioration in Anne Elliot’s appearance can, in fact, be illustrated with the words of Wiltshire and Wiltshire, who claim that the protagonist “wears her sadness and deprivation in her prematurely aging body and face” (Wiltshire 1992, 155). And then in the mouth of Anne herself, who muses that “[h]er attachment and regrets had, for a long time, clouded every enjoyment of youth; and an early loss of bloom and spirits had been their lasting effect” (Austen 2009, 25). Through this remark, readers are introduced to the main reason that stands behind her haggard physique: the fact that, eight years ago, *persuaded* by her family, she did not continue her relationship with Captain Wentworth as he was not particularly well-off at the time.

Anne explicitly makes the connection between having had to abandon the pursuit of her crush and her loss of youth when she thinks to herself that the decision put her “[i]nto a state of most wearing, anxious, youth-killing dependence!” (Austen 2009, 24). In this train of thought, the “loss of bloom” is not only described as having had physical consequences but psychological too, since she refers to this event as having put her in a “state” of weariness and anxiety, which in themselves can simultaneously be seen as causes of physical decline (“youth-killing dependence”). Austen, however, also points to the influence that Anne’s personality had on this process since the protagonist believes herself always “to struggle against a great tendency to lowness” (Austen 2009, 87). This is a reflection that Sara Tavela identifies as “Austen’s definition of psychosomaticism,” who then points to Lady Russell’s remark that Anne’s spirits were, frequently, “not high” (Tavela 2017, 177–78).

Based on that evidence, Tavela has concluded that the protagonist of *Persuasion* suffers “from what we now would term depression” (Tavela 2017, 179). Although, as it was discussed above, depression is a condition that has been identified as a causal factor in early senescence, depressive processes can be explained by multiple factors other than a relationship breakdown. That is why it may be more adequate to read Anne’s symptoms as indicators of psychological distress but not depression. Similarly, accelerated aging could also be considered from other influences present in Austen’s time, such as moral treatment or even from the perspective of other contemporary paradigms, including Trauma Informed Care.

Moral treatment arose as a response to mental disorders and was grounded in compassionate psychosocial care and ethical discipline. Originating in the eighteenth century, this approach gained prominence during the nineteenth century, drawing influence from psychiatry and psychology, as well as religious and moral considerations. According to Crossley (2006), this movement played a significant role in the reform and advancement of the asylum system in Western Europe during this period. Moral treatment emphasized factors such as emotions and the ethical components of social interactions to the detriment of biological elements.

From this perspective, *Persuasion* can be seen as a reflection of the importance of ethical behavior and the consequences of disregarding one’s moral compass. Throughout the story, we witness the consequences of persuasion and manipulation. Characters like Sir Walter Elliot and Mrs. Clay exemplify the negative effects of prioritizing self-interest over moral principles. Their actions lead to personal dissatisfaction and strained relationships. On the other hand, the protagonist, Anne Elliot, embodies the virtues of moral treatment. Despite being persuaded to break off her engagement with Captain Wentworth years ago, she remains steadfast in her love and loyalty. In this novel, Austen subtly reminds readers of the importance of moral behavior and the potential pitfalls of succumbing to societal pressures or personal gain.

For its part, Trauma Informed Care (TIC) is a conceptual framework aimed at effectively supporting individuals who have encountered adverse outcomes following exposure to hazardous events. TIC principles prioritize a comprehensive view of what encompasses psychological turmoil and how ensuing trauma affects various aspects of human well-being, including physical health, cognition, emotions, actions, interpersonal interactions, and social connections (Evans and Coccoma 2014). Most TIC frameworks adopt a biopsychosocial standpoint, acknowledging the interrelated impacts on biological functions (body and brain), psychological states (mind), and interpersonal dynamics (relationships). Exposure to stressful circumstances is believed to influence an individual’s previous and current adaptive responses, as well as their patterns of information processing (Evans and Coccoma 2014, 22).

From a TIC perspective, one can interpret Anne’s experiences through the lens of trauma and its impact on her emotional well-being. Anne faces the trauma of societal expectations, familial pressures, and the consequences of a decision made under external influence. In Anne’s case, TIC involves recognizing the emotional toll of her past experiences and acknowledging the impact that societal norms have had on her decisions. As Anne and Captain Wentworth navigate their renewed connection once they meet again, TIC would underscore the importance of open communication, trust-building, and creating a space for Anne to express her emotions without judgment. The novel’s resolution could be seen as a testament to the healing power of understanding and addressing past traumas in the context of intimate relationships and personal growth.

Resuming the discussion of the protagonist’s appearance, Anne’s deterioration also becomes reflected on her face and body. Thinking about how she looks in the eyes of the Captain the first time they see each other after all those years, she notices how he observes “[h]er altered features, perhaps, trying to trace in them the ruins of the face which had once charmed him” (Austen 2009, 64). The hyperbolic use of the word “ruins” suggests that Anne’s face is in a state of decay, which most surely constitutes a reference to her having developed fine lines as well as a cranky look.

Quite promptly in the novel, readers are also informed of Anne’s wasted physique:

A few years before, Anne Elliot had been a very pretty girl, but her bloom had vanished early; and as even its height, her father had found little to admire in her, (so totally different were her delicate features and mild dark eyes from his own); there could be nothing in them now that she was faded and thin, to excite his esteem. (Austen 2009, 6).

Through her father’s derogatory opinion, Anne’s premature aging can be observed in her generally old and sunken (“faded”) features, as well as in her marked weight loss. This latter aspect is insisted upon when Anne’s body is referred to as having developed “a slender form” (Austen 2009, 60). Insistence on this indicates that Jane Austen intuitively associated psychological turmoil with weight loss. This is an aspect that has been confirmed today by psychoneuroimmunology and that we have discussed earlier on, but which had already been anticipated in the Romantic’s intuitive connection established between lovesickness and weight loss. In this regard, Gorman documents that “Austen knows the clear connection between the mental and the physical that eighteenth-century doctors were beginning to document” since “[e]arly in the novel, Jane’s colds and poor appetite were noted by her relatives,” this latter symptom being “associated with stress and with lovesickness” (Gorman 1990, 130). Annette Upfal also notes that “a connection between nervous illness and digestive symptoms was generally known and accepted” (Upfal 2014, 156), pointing to how Trotter linked weight and appetite loss to “grief and disappointed love” (210).^1^

While the beginning of *Persuasion* focuses on Anne’s prematurely aging physique in quite literally descriptive terms, later in the novel, references to this same topic are captured in more figurative and indirect ways. On the one hand, references to the passage of time and the seasons are made to symbolize human decay. For example, at one point, the narrator recalls a “tender sonnet, fraught with the apt analogy of the declining year, with declining happiness, and the images of youth and hope, and spring, all gone together” (Austen 2009, 75). Todd and Blank identify Anne’s tender sonnet as a reference to Charlotte Smith’s “Sonnet 2,” from the *Elegiac Sonnets*, a poem the protagonist would remember in an attempt to put her mind off the Captain’s romantic advancements with Louisa. Lines in the sonnet include: “Poor humanity! So frail, so fair / Are the fond visions of thy early day, / Till tyrant passion and corrosive care / Bid all thy fairy colours fade away! / Another May new buds and flowers shall bring / Ah! Why has happiness—no Second spring!” For us, the two references made to the triumph of winter over spring point more generally to the Latin motifs of *carpe diem* and *collige virgo rosas*. These motifs would have been employed here to emphasize the feelings of resentment and regret that Anne experiences as a result of having succumbed to her family’s pressures to abandon the relationship with the Captain in the past.

Continuing with references made to the cold months of the year, it is significant that twice autumn is mentioned in the book in the context of Anne’s role as a caregiver. The first time, it is Anne who, somewhat passively, muses that:

Mary, often a little unwell, and always thinking a great deal of her own complaint, and always in the habit of claiming Anne when any thing was the matter, was indisposed; and foreseeing that she should not have a days health all the autumn, entreated, or rather required her, for it was hardly entreaty, to come to Uppercross cottage and bear her company as long as she should want her, instead of going to Bath. (Austen 2009, 29).

And then, near the end of the book, Mary talks once more about “keeping Anne with her in the autumn” (Austen 2009, 217). In short, the family’s assumptions of Anne’s nursing duties entrap her for the autumn season on several occasions, putting her in a perennial state of having to subordinate her own needs to those of others, which is what the psychosocial perspective of moral treatment would essentially encompass.

Another symbolic reference to the passage of time is made when Anne addresses the consequences of the flow of life on the house furnishings. In referring to her sister, Anne insists that Mary was “now lying on the faded sofa of the pretty little drawing-room, the once elegant furniture of which had been gradually growing shabby, under the influence of four summers and two children” (Austen 2009, 32). Her allusion to the seasons and children having a youth-killing effect on furniture indirectly points to one explanation for why Anne appears older than her age, for she is the one who is also put in charge of little Charles when he becomes sick. We have already pointed to the connection between sick care of children and premature aging but also to the main aspects of moral treatment theory, which helps to explain Anne’s moral behavior in the context of societal expectations about women and infant care.

Expanding on the furniture motif, when the Captain is considering renting Elliot’s house and the family worries about necessary improvements that must be made to it, the narrator focuses on how they needed “to supply the deficiencies of lodging house furniture, and defend the windows and doors against the winter storms to be expected” (Austen 2009, 88). Mentioning the weather inclemency of the wintery session is a way of making a reference to how the passage of time deprives entities, both human and material, of beauty and, therefore, of (marriage) market value. In this regard, we agree with Wiltshire, who draws the connection between the protagonist’s declining body and the narrator’s focus on furniture decay when he claims that, in *Persuasion*, “[t]he body is perceived as an object; it’s to be prized or appraised, like handsome furniture, as a commodity” (Wiltshire 1992, 161).

Yet *Persuasion* is a novel with a happy ending. In an accelerated turn of events, Anne understands that the Captain still feels a great sense of affection for her, and upon receiving a letter from him in which he declares his love for her, feelings of recovered love and happiness start to show on the protagonist’s face:

Anne saw nothing, thought nothing of the brilliancy of the room. Her happiness was from within. Her eyes were bright, and her cheeks growled but she knew nothing about it. She was thinking only of the last half hour, and as they passed to their seats her mind took a hasty range over it. (Austen 2009, 163).

Gorman clarifies the parallelism between Anne’s loss and regained bloom caused by disappointed and recovered love, respectively, when she claims that “[t]he recovery of love is so important to the well-being of Anne Elliot that the blood, the life forces, animates her face as Wentworth returns to her emotional life” (Gorman 1990, 248).

Furthermore, just as weight loss had been associated with Anne Elliot’s lovesickness and gloomy feelings, weight gain is revealed as a consequence of the protagonist’s improved mood when she first regained contact with the Captain. At that point, the narrator announced that “Anne was improved in plumpness and looks,” drawing a connection between a certain degree of physical robustness and a generally good-looking appearance (“looks”), as her physique aroused in Anne the feeling “that she was to be blessed with a second spring of youth and beauty” (Austen 2009, 107). In this regard, Gorman notes that “[c]onnections between good health and good looks, common enough in the twentieth century” varied significantly during the Romantic era (Gorman 1990, 253). In particular, she claims that:

Late eighteenth-century observers not only observed empirically the natural connection between health and good looks; they also had inherited the pseudo-scientific movement of physiognomy, which culminated in the works of Johann Kaspar Lavater … who posited a close connection between appearance and robustness by plumpness. (Gorman 1990, 253–254).

The reference to the works of Johan Kaspar Lavater is relevant since he was a contemporary of Jane Austen, and both wrote before the post-Romantic admiration of tubercular beauty, which praised the sickly look of tuberculosis.

At the end of *Persuasion*, the narrator closes the connection between Anne recovering the Captain’s affections and her improved looks when she describes the following scene: “Anne smiled, and let it pass. … It is something for a woman to be assured, in her eight-and twentieth year, that she has not lost one charm of her earlier youth: but the value of such homage was inexpressibly increased to Anne,” who felt it “to be the result, not the cause of a revival of his warm attachment” (Austen 2009, 212). The quote shows once more that Austen seemed cognizant of the relationship between a healthy affective and psychological state and physical vigor and good appearance.

### Jobs and physical appearance

In the previous sections, the relationship between mind, emotions, and body has been discussed, as well as how Austen was able to intuit the effects of this relationship. Austen instinctively understood that premature aging, or at least the loss of bloom, could be the consequence of disappointed love. However, she also perceived that this was not the only cause, but it could be due to external factors of various kinds, as can be seen in *Persuasion*, which is undoubtedly the novel in which the author addresses the psychosocial repercussions of physical appearance the most (Warhol 1992).

In chapter 3 of this novel, we find a dialogue in which Sir Walter Elliot shows a contemptuous attitude towards the navy. Although he does not deny the need for this profession, he states that he would not recommend it to any of his friends for two reasons. The first, in Sir Walter Elliot’s words, the navy had become “the means of bringing persons of obscure birth into undue distinction” (Austen 2009, 17). That is to say, it allowed some men of humble origin to ascend socially. The second reason, which is the one that is most related to our work, is the harmful consequences of this job for the health of those who perform it, “as it cuts up a man’s youth and vigour most horribly; a sailor grows old sooner than any other man” (17).

To illustrate his words, Sir Walter recounts a personal memory of a naval officer he was introduced to some years earlier, whom he describes as “the most deplorable-looking personage you can imagine; his face the colour of mahogany, rough and rugged to the last degree; all lines and wrinkles” (Austen 2009, 17). Sir Walter contemptuously describes the physical deterioration of this naval officer to whom he attributed the age of sixty or sixty-two when he was only forty, which leads him to conclude:

I shall not easily forget Admiral Baldwin. I never saw quite so wretched an example of what a sea-faring life can do; but to a degree, I know it is the same with them all: they are all knocked about, and exposed to every climate, and every weather, till they are not fit to be seen. (Austen 2009, 18).

The dialogue we are commenting on continues with Mrs. Clay’s response, who considers that all jobs negatively affect health, be it the army, the law, medicine, or even the church. For this reason, she affirms that although all professions are necessary and honorable in their own way, they harm the health and physical appearance of those who carry them out. Physical well-being is, therefore, the heritage of only a privileged few:

[I]t is only the lot of those who are not obliged to follow any, who can live in a regular way, in the country, choosing their own hours, following their own pursuits, and living on their own property, without the torment of trying for more; it is only their lot, I say, to hold the blessings of health and a good appearance to the utmost. (Austen 2009, 18).

As we pointed out earlier, this reflects that good health and physical attractiveness are to some extent related to social position and economic status and that health, in Austen’s time, was a luxury that should not be risked, especially in the case of women, since any impairment in beauty could affect the value of a young woman in the marriage market.

In *Persuasion*, however, we find a striking contrast in the way both disappointments in love and the passage of time affect the novel’s two main characters. In the case of Anne Elliot, it can be seen how, from the beginning, her physical deterioration is highlighted due to the suffering caused by the breakup of her engagement. However, when Captain Wentworth is mentioned, it is always done in a laudatory tone, to praise his vigor and good looks despite the passage of time, having suffered the same disappointment as Anne, and having been exposed to the inclemency of weather on his sea voyages. In fact, Sir Walter Elliot himself, so critical of the navy, does not hesitate to show his admiration for the attractiveness of Captain Wentworth in a conversation:

“A well-looking man,” said Sir Walter, “a very well-looking man.”

“A very fine young man indeed!” said Lady Dalrymple. “More air than one often sees in Bath. Irish, I dare say.”

“No, I just know his name. A bowing acquaintance. Wentworth; Captain Wentworth of the navy.” (Austen 2009, 165).

The fact that Austen describes Captain Wentworth so favorably may be due in part to her idealized image of the navy, as a result of two of her brothers being naval officers (Upfal 2005, 185). In addition, Captain Wentworth’s attractiveness is used by Austen to create a greater contrast between him and Anne Elliot. Captain Wentworth acknowledges the evident difference between the two, as shown in the passage in which Henrietta Musgrove comments to Anne that Captain Wentworth had said that he had found her so changed that he would not have recognized her, which provokes the following reflections from the protagonists:

“Altered beyond his knowledge.” Anne fully submitted, in silent, deep mortification. Doubtless it was so, and she could take no revenge, for he was not altered, or not for the worse. … No: the years which had destroyed her youth and bloom had only given him a more glowing, manly, open look, in no respect lessening his personal advantages. She had seen the same Frederick Wentworth. (Austen 2009, 54).

In this way, readers can appreciate the different attitudes of the two protagonists in the face of the same event and, by extension, the very different ways in which men and women face the consequences of disappointed love. This idea is later reinforced when Anne and Captain Wentworth meet again in Bath after announcing the engagement between Captain Benwick and Louisa Musgrove, with whom Captain Wentworth seemed in love and, observing him carefully, Anne concludes that “He looked very well, not as if he had been suffering in health or spirits” (Austen 2009, 154).

Austen addresses through these examples an issue that was current in her contemporary society and literature, the contrast between the supposed inconstancy in women and the praised persistence in men. In fact, this is the subject of a conversation between Anne Elliot and Captain Harville:

“I believe in a true analogy between our bodily frames and our mental; and that as our bodies are the strongest, so are our feelings; capable of bearing most rough usage, and riding out the heaviest weather.”

“Your feelings may be the strongest,” replied Anne, “but the same spirit of analogy will authorise me to assert that ours are the most tender. Man is more robust than woman, but he is not longer lived; which exactly explains my view of the nature of their attachments.” (Austen 2009, 203–204).

As can be seen, through the male character Austen creates the analogy between bodily strength and strength of feelings, which contrasts with what the readers of the novel perceive, that is, the constancy of Anne Elliot’s love for Captain Wentworth and his seeming indifference to her; indifference that is a mask to cover a feeling of wounded pride. In other words, the different physical consequences of their love and breakup represent the attitude of each of the characters in the novel.

This contrast also reflects the stereotyped belief of the time, according to which time positively affected men since wrinkles and grey hair gave them character, while, in the case of women, age not only affected fertility but also caused a loss of physical attractiveness. In addition, another highly relevant difference is that men had occupations that forced them to travel, to have goals, and to relate to a variety of people so that it was easier for them to overcome emotional suffering. The lives of women, however, lacked this type of stimuli, so the melancholy was aggravated, presumably producing a much more pronounced physical and emotional decline. In *Persuasion*, we find a passage in which Anne Elliot makes this contrast manifest:

We certainly do not forget you as soon as you forget us. It is, perhaps, our fate rather than our merit. We cannot help ourselves. We live at home, quiet, confined, and our feelings prey upon us. You are forced on exertion. You have always a profession, pursuits, business of some sort or other, to take you back into the world immediately, and continual occupation and change soon weaken impressions. (Austen 2009, 203).

Based on this reflection and on the role of the protagonist for much of the novel, Tavela states that “Anne anticipates the crux of Victorian psychosomatic heroines’ difficulties” (Tavela 2019, 13). In this way, it is revealed how the Victorian obsession with the special susceptibility to psychological suffering of women contrasts, in reality, with the cause of the same. Following Elaine Showalter in her seminal work *The Female Malady,* women’s psychological turmoil would be the result of their daily lives because the “suffocation of family life, boredom, and patriarchal protection gradually destroys women’s capacity to dream, to work, or to act” (Schowalter 1987, 61).

## Conclusions

In this article, we have approached the connection between literature, medicine, and psychology from an innovative perspective by assessing Jane Austen’s novel *Persuasion* from a psychoneuroimmunological perspective and, thus, delving into the relationship between emotional and mental health and physical appearance. This research has revealed how Austen’s ability to observe and her profound perception of human psychology allowed her to intuit the relationship between disappointment in love, depressive processes, and premature aging.

Through the character of Anne Elliot, Austen exemplifies in a paradigmatic way how a strong emotional setback, such as the breakup of a romantic relationship due to social pressure, can cause a loss of bloom in a young woman and, conversely, how emotional recovery is accompanied by a process of physical rejuvenation. These facts, as has already been mentioned, reflect processes that the discipline of psychoneuroimmunology has not been able to decipher until practically today. In this regard, a limitation of the study is that this is a modern conception that is being used to explain Austen’s picture of the heroine, which nonetheless could also be accounted for by other theories, including earlier ones, such as moral treatment, or other contemporary paradigms, including Trauma Informed Care.

The decision to place most of the focus of literary analysis on the character of Anne Elliot is not only due to her role as the protagonist of *Persuasion*, which is the novel we chose for our research. Rather, from a gender point of view, this book illustrates some of the latest discoveries in psychoneuroimmunology by representing how depressive processes seem to cause more intense premature aging in women than in men. Austen, through the contrast between Anne Elliot and Captain Wentworth, showed how the different social habits of men and women, apart from underlying biological differences, affected their health with various results. Women’s confinement in the home and the burden of the role of caretaker, as well as the absence of activity and vital goals, had as a consequence their greater propensity towards melancholy and depression and, therefore, to premature aging. Conversely, these maladies were less frequent in men, whose lives were not subject to so many limitations and given potential for expansion through work outside the home. It is hoped that this article has been able to capture that Austen anticipated a proto-feminist viewpoint and how, inadvertently or not, gave voice to the concerns of a woman that are relevant for the discipline of psychoneuroimmunology in a way that we are only now in the process of (re)interrogating.
